# Prospective, Single-Arm, Investigator-Initiated Study to Evaluate Efficacy and Safety of Levocetirizine Hydrochloride 5 mg and Montelukast 10 mg (L-Montus) in Patients With Seasonal Allergic Rhinitis and Perennial Allergic Rhinitis

**DOI:** 10.7759/cureus.104156

**Published:** 2026-02-23

**Authors:** Chidananda Ramappa, Manoj Kumar Gola, Karthikeyan Padmanabhan, Gayatri Veeramani Jayaraman, Ashwin Karuppan, Aafrin Shabbir, Hariharan S

**Affiliations:** 1 Otorhinolaryngology, Medstar Speciality Hospital, Bangalore, IND; 2 Otorhinolaryngology, Udyaan Healthcare, Lucknow, IND; 3 Otorhinolaryngology, Mahatma Gandhi Medical College and Research Institute, Puducherry, IND; 4 Pulmonology, Fourrts India Laboratories, Chennai, IND; 5 Internal Medicine and Diabetology, Gleneagles Global Health City, Chennai, IND; 6 Internal Medicine and Diabetology, Tambaram Medical Center, Chennai, IND; 7 Pharmacology, Tambaram Medical Center, Chennai, IND

**Keywords:** antihistamines, fixed-dose combination, levocetirizine, montelukast, seasonal allergic rhinitis

## Abstract

Introduction

To evaluate the efficacy and safety of a fixed-dose combination (FDC) of levocetirizine hydrochloride 5 mg and montelukast 10 mg (L-Montus) in the treatment of seasonal allergic rhinitis (SAR) and perennial allergic rhinitis ( PAR ) among adult patients in India.

Methods

This was a prospective, single-arm, multicenter clinical study conducted across three clinical sites in India. A total of 200 patients aged 18 to 64 years with moderate-to-severe SAR were enrolled. All patients received once-daily oral doses of the FDC for eight weeks, with follow-up extending to 13 weeks. Symptom severity was assessed using validated tools: total nasal symptom score (TNSS), total ocular symptom score (TOSS), and night-time symptom score (NTSS). Safety and treatment adherence were monitored throughout the study. Statistical significance was evaluated using the Friedman and Wilcoxon signed-rank tests.

Results

At baseline, nasal symptoms such as sneezing (100%), rhinorrhea in 194 patients (97%), and nasal congestion in 191 patients (95.5%) were highly prevalent. By week eight, TNSS decreased from 6.74±2.28 to 0.86±1.11 (p=0.005), TOSS from 5.60±2.44 to 0.61±0.87 (p=0.005), and NTSS from 4.99±2.28 to 0.37±0.59 (p=0.005), demonstrating significant symptom relief. The treatment was well-tolerated, with only 14% (28) of patients reporting mild adverse events; no serious adverse events or discontinuations were observed.

Conclusions

The FDC of levocetirizine and montelukast (L-Montus) demonstrated robust efficacy in reducing nasal, ocular, and nocturnal symptoms associated with SAR, with a favorable safety and adherence profile. The dual mechanism of action targeting histaminergic and leukotriene pathways likely contributed to the comprehensive symptom control. These findings support the FDC as an effective first-line therapeutic option in the Indian SAR population.

## Introduction

Allergic rhinitis (AR) is a prevalent chronic condition characterized by immune-mediated inflammation of the nasal mucosa following exposure to airborne allergens [1]. Globally, it affects a substantial proportion of the population-estimates suggest that approximately 10-30% of adults and up to 40% of children suffer from this condition [2]. AR often presents with a constellation of symptoms, including nasal congestion, sneezing, itching, rhinorrhoea, and may also involve ocular manifestations [3]. These symptoms not only impair quality of life but also interfere with daily functioning, sleep, academic performance, and productivity, making AR a significant public health concern [4,5].

The allergic rhinitis and its impact on asthma (ARIA) guidelines classify AR based on its chronicity, either intermittent or persistent, and on the severity of its impact on the patient’s quality of life (mild or moderate/severe) [3]. Traditionally, allergic rhinitis is also subdivided into seasonal (SAR), often triggered by pollens during specific times of the year, and perennial (PAR), which persists throughout the year due to continuous exposure to allergens such as dust mites, animal dander, or mold spores [6].

Management of AR involves a symptom-guided approach with pharmacologic therapy forming the cornerstone of treatment [7]. The therapeutic arsenal includes oral and intranasal H1-antihistamines, intranasal corticosteroids, leukotriene receptor antagonists (LTRAs), decongestants, and other adjunctive agents [8]. Among these, H1-antihistamines are considered the first-line agents, especially in cases with mild-to-moderate disease, due to their efficacy in controlling sneezing, nasal itch, and rhinorrhoea. However, they are less effective in managing nasal congestion [9].

First-generation antihistamines are associated with central nervous system side effects, including sedation and impaired cognitive performance, limiting their use [10]. In contrast, second-generation antihistamines, such as levocetirizine, offer a favorable safety profile with minimal sedative effects and improved patient tolerability [11]. Levocetirizine, a pharmacologically active R-enantiomer of cetirizine, exerts its therapeutic effect by blocking peripheral histamine H1-receptors. Clinical studies have supported its role in mitigating symptoms of both seasonal and perennial allergic rhinitis while maintaining a favorable safety and tolerability record [12, 13].

Montelukast is an orally active leukotriene receptor antagonist that selectively binds to the cysteinyl leukotriene type 1 (CysLT1) receptor. By inhibiting the action of leukotriene D4, montelukast helps reduce inflammation and nasal congestion, particularly during the night [14]. While montelukast can independently alleviate allergic rhinitis symptoms, it is frequently prescribed in combination with antihistamines to enhance symptom relief, particularly for cases involving ongoing or nighttime nasal obstruction [15-17].

Several studies have indicated that the combined use of an H1-antihistamine with an LTRA may provide enhanced symptom control compared to monotherapy [18-21]. Specifically, the fixed-dose combination of levocetirizine 5 mg and montelukast 10 mg has demonstrated superior efficacy in alleviating both daytime and nighttime symptoms in patients with SAR and PAR [22]. The combination therapy is generally well tolerated and addresses a broader spectrum of AR symptoms, making it a valuable therapeutic option in clinical practice [23].

In this context, the present study was designed as a prospective, single-arm, investigator-initiated study to evaluate the efficacy and safety of the fixed-dose combination of levocetirizine and montelukast in Indian patients diagnosed with seasonal or perennial allergic rhinitis. The primary objective of this study was to evaluate the efficacy of the fixed-dose combination in reducing symptom severity as measured by Total Nasal Symptom Score (TNSS). Secondary objectives included assessment of ocular symptoms (TOSS), nighttime symptoms (NTSS), safety profile, and treatment adherence. This study focused on symptom control and did not include objective biomarkers or measures of inflammatory regression.

## Materials and methods

Study design and oversight

This study was a prospective, multicenter, single-arm study conducted to evaluate the efficacy and safety of a fixed-dose combination of levocetirizine hydrochloride 5 mg and montelukast 10 mg (L-Montus) in patients with seasonal allergic rhinitis (SAR). The study was designed to assess symptom control rather than disease regression or cure. The study included both male and female participants aged 18-65 years and spanned a total study duration of 13 weeks, including an 8-week treatment phase and a follow-up period extending to week 13. Schedule of Study Assessments and Timepoints Across Visits presented in Table 1

The study was conducted at three clinical sites in India: Mahatma Gandhi Medical College and Research Institute (Pondicherry), Medstar Speciality Hospital (Bangalore) and Udyaan Healthcare (Lucknow). Site 1 (Health Point Hospital, Kolkata) was approved for participation but did not enrol patients due to delayed ethics approval.

The study followed ethical standards as outlined in the Declaration of Helsinki (Taipei 2016), ICH-GCP guidelines, the New Drugs and Clinical Trials Rules (2019), and Schedule Y (schedule in the Drugs and Cosmetics Act of India). Institutional Ethics Committees at each site reviewed and approved the protocol and informed consent documents. Written informed consent was obtained from each participant before study initiation. Study monitoring and data management were performed by IDD Research Solutions Pvt. Ltd., Bangalore.

The study evaluated clinical efficacy based on validated patient-reported symptom scores (TNSS, TOSS, NTSS). Objective measures of inflammatory activity or structural disease regression, such as nasal endoscopy, serum IgE levels, eosinophil counts, or rhinomanometry, were not included, as the primary focus of the study was symptomatic improvement consistent with routine clinical practice.

Patients and study measurements

A total of 200 patients were enrolled and completed the study. All patients received a once-daily oral dose of the fixed-dose combination tablet (levocetirizine 5 mg + montelukast 10 mg) at bedtime for eight weeks.

Inclusion criteria required patients to have moderate-to-severe intermittent or mild persistent SAR, a Total Nasal Symptom Score (TNSS) ≥5 [24], and no antihistaminic use in the preceding week. Exclusion criteria included hypersensitivity to the study drugs, comorbid respiratory or psychiatric conditions, recent nasal surgeries, and concurrent use of other treatments for allergic rhinitis. No additional rescue medications were permitted during the study period. The schedule of assessments is summarized in Table 1 below.

**Table 1:** Schedule of Study Assessments and Timepoints Across Visits. The table indicates that the respective assessment or procedure was conducted at the specified visit. Telephone (Tel.) visits at weeks 2, 4, and 13 were conducted remotely. Global assessment was performed by both patient and physician at the end-of-study visit (week 13). TNSS: total nasal symptom score; TOSS: total ocular symptom score; NTSS: night-time symptom score.

Study Procedure	Visit 1 (Day 1)	Visit 2 (Week 2, Tel.)	Visit 3 (Week 4, Tel.)	Visit 4 (Week 8)	Visit 5 (Week 13, Tel.)
Informed Consent	✔				
Inclusion/Exclusion Criteria	✔				
Medical History	✔				
Physical Examination	✔			✔	
Vital Signs	✔			✔	
Concomitant Medications	✔	✔	✔	✔	✔
Rhinitis Symptoms (TNSS, TOSS, NTSS)	✔	✔	✔	✔	✔
Adverse Events	✔	✔	✔	✔	✔
Global Assessment (Patient & Physician)					✔

Rhinitis symptoms were assessed using validated 4-point Likert scales. The total nasal symptom score (TNSS) evaluated rhinorrhea, nasal congestion, nasal itching, and sneezing [24]. The total ocular symptom score (TOSS) assessed tearing, pruritus, redness, and puffiness [25]. The night-time symptom score (NTSS) evaluated difficulty sleeping, night-time awakenings, and nasal congestion upon awakening [26]. Global assessments of efficacy and tolerability were conducted at Week 13 by both investigator and patient using 4-point and 3-point categorical scales, respectively. Global assessments of efficacy and tolerability were predefined and conducted at the end-of-study visit (Week 13) by both the investigator and the patient using categorical rating scales.

Treatments and adherence

Patients received study medication with instructions for daily oral administration at bedtime with ambient temperature water. Adherence was monitored via returned tablet counts and patient diaries, which recorded daily dosing details. Investigators documented dosing adherence in the case report forms (CRFs).

Safety assessments

All adverse events (AEs), serious adverse events (SAEs), adverse drug reactions (ADRs), and unexpected AEs/SAEs were recorded. Safety evaluations included physical examinations and vital sign measurements during clinic visits. AEs were categorized by severity and assessed for causality.

Statistical analysis

As an exploratory, descriptive study, no formal hypothesis testing was performed. Continuous variables (e.g., symptom scores) were summarized using mean, standard deviation, median, minimum, and maximum. Categorical variables (e.g., adverse event frequencies) were summarized using counts and percentages.

Efficacy analyses were conducted on both the intent-to-treat (ITT) and per-protocol (PP) populations, while the safety population included all subjects who received at least one dose of study medication. TEAEs (Treatment-emergent adverse events) were coded according to WHOART terminology and categorized by system organ class, preferred term, severity, and relationship to study drug. Treatment adherence and efficacy variables, including TNSS, TOSS, and NTSS, were analyzed using appropriate statistical methods (Friedman test and Wilcoxon signed-rank test).

## Results

Subject demographics

A total of 200 patients were enrolled in the study and received at least one dose of the fixed-dose combination of levocetirizine hydrochloride 5 mg and montelukast 10 mg. The gender distribution was relatively balanced, with 105 (52.5%) male and 95 (47.5%) female participants. The mean age of the study population was 36.8 years. The age range of participants was 18 to 64 years, reflecting a broad adult population with seasonal allergic rhinitis. All enrolled subjects were of Asian ethnicity (100%), consistent with the study's conduct across multiple Indian clinical sites. The basic demographic characteristics are summarized in Table 2. All 200 enrolled patients completed the study and were included in the final analysis. No patient discontinued due to adverse events or protocol deviations. Concomitant medications for allergic rhinitis were not permitted during the study period.

**Table 2:** Summary Table of Efficacy Symptoms (ITT population, N=200). Symptoms were assessed based on patient self-report using a 4-point Likert scale: 0=not noticeable, 1=mild, 2=moderate, 3=severe. Responses reflect the presence or absence of symptoms as reported at baseline (Visit 1) by the intent-to-treat (ITT) population (N=200). The use of a patient-reported scale ensured credibility and patient-centred evaluation of symptom severity. Percentages represent the proportion of participants reporting each symptom.

Symptom	Response	Frequency (percentage)
Nasal congestion	Yes	191 (95.5 %)
Nasal congestion	No	09 (4.5 %)
Rhinorrhea	Yes	194 (97 %)
Rhinorrhea	No	6 (3 %)
Nasal itching	Yes	176 (88 %)
Nasal itching	No	24 (12 %)
Sneezing	Yes	200 (100 %)
Sneezing	No	0 (0 %)
Itchy or burning eyes	Yes	98 (49 %)
Itchy or burning eyes	No	102 (51 %)
Teary or watery eyes	Yes	180 (90 %)
Teary or watery eyes	No	20 (10 %)
Redness in eyes	Yes	174 (87 %)
Redness in eyes	No	26 (13 %)

Measurements of treatment adherence

All patients were given enough quantity of study medication as per protocol. They were instructed to maintain their daily dose details in the patient diary. Subject data were reviewed, along with drug accountability at the clinic visit (Visit 4-week 8), to assess treatment compliance. The subjects were followed up by the investigator for signs and symptoms and adverse events (AEs) until week 13. The medical adherence was determined from the returned tablet count. Details of dosing were documented appropriately in the CRF.

Treatment adherence

Treatment adherence, assessed through returned tablet count and diary entries, was high throughout the study, with more than 95% of prescribed doses taken. No patient discontinued treatment due to poor adherence.

Analysis of efficacy result

At baseline, the prevalence of allergic rhinitis symptoms was high among the enrolled patients (N=200), as summarized in Table 2 above. Patients self-rated their symptoms using a 4-point Likert scale ranging from 0 (not noticeable) to 3 (severe), which ensured the credibility and patient-centeredness of symptom assessment. The most commonly reported nasal symptoms included sneezing, nasal congestion, rhinorrhea, and nasal itching. All patients (100%) reported experiencing sneezing, while 191 patients (95.5%) experienced nasal congestion, and 194 (97%) reported rhinorrhea. Nasal itching was reported by 176 patients (88%). Ocular symptoms were also prominent. Teary or watery eyes were reported by 180 patients (90%), and redness in the eyes by 174 patients (87%). Itchy or burning eyes were present in 98 patients (49%). These findings indicate a substantial burden of both nasal and ocular symptoms in the study population at entry.

Symptom reduction over time

The efficacy of the fixed-dose combination (FDC) of levocetirizine hydrochloride 5 mg and montelukast 10 mg was assessed using three validated symptom scales: Total nasal symptom score (TNSS), total ocular symptom score (TOSS), and night time symptom score (NTSS). Each was measured across five time points from baseline (Visit 1, Day 1) to the end of the study (Visit 5, week 13), capturing nasal, ocular, and nighttime symptoms associated with seasonal allergic rhinitis.

Total nasal symptom score (TNSS)

At baseline (Visit 1), the mean TNSS was 6.74 ± 2.28, with a median score of 6, indicating moderate to severe nasal symptoms including rhinorrhea, nasal congestion, nasal itching, and sneezing. Over the course of treatment, a consistent and substantial reduction in TNSS was observed, reaching a mean of 0.86 ± 1.11 and a median of 0 by Visit 5. The range of TNSS scores declined from 3-12 at baseline to 0-5 at the end of the study, demonstrating a marked reduction of nasal symptoms in most patients. Statistical analysis using the Friedman test indicated that the reduction in TNSS across visits was statistically significant (p = 0.005). Pairwise comparisons using the Wilcoxon signed-rank test confirmed significant improvement from Visit 1 to each subsequent visit (V2, V3, V4, and V5), also at p=0.005.

Total ocular symptom score (TOSS)

The baseline mean TOSS was 5.60 ± 2.44, with a median of 5 and a score range of 0-12, reflecting the presence of symptoms such as tearing, pruritus, redness, and puffiness of the eyes. By Visit 5, the mean score had declined to 0.61 ± 0.87, with a median of 0 and a range of 0-6. This reduction indicates substantial improvement in ocular symptoms over time with continued treatment. The Friedman test showed a statistically significant change in TOSS scores across all visits (p=0.005), and pairwise Wilcoxon tests confirmed that symptom reductions from baseline to each follow-up visit were also statistically significant (p=0.005).

Night-time symptom score (NTSS)

NTSS, calculated as the sum of three individual nighttime symptoms (difficulty falling asleep, nighttime awakenings, and nasal congestion upon awakening), also showed marked improvement. At Visit 1, the mean NTSS was 4.99 ± 2.28, with a median of 5 and a range of 0-9. By Visit 5, the mean had decreased to 0.37 ± 0.59, with a median of 0 and a range of 0-3, signifying that patients experienced significantly less night-time disruption as treatment progressed.

**Figure 1:**
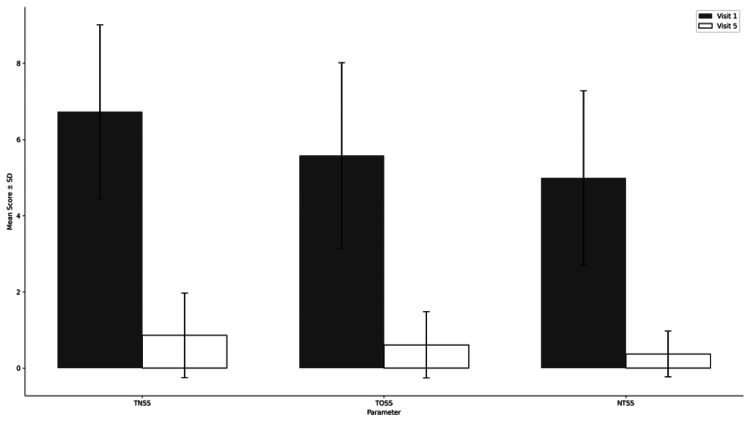
Efficacy Analysis Based on TNSS, TOSS, and NTSS Scores Over Five Visits.

This improvement was again supported by a statistically significant Friedman test result (p=0.005). Pairwise comparisons between baseline and each follow-up visit using the Wilcoxon signed-rank test were all statistically significant (p = 0.005), further confirming the treatment’s efficacy in alleviating nighttime symptoms. The summary of efficacy analysis is presented in Table 3 and Figure 1.

**Table 3:** Summary of Efficacy Analysis Based on TNSS, TOSS, and NTSS Scores Over Five Visits (Friedman Test). All values are expressed as mean ± SD. Statistically significant reductions were observed in all symptom scores between Visit 1 and Visit 5 using the Friedman test (p<0.005). TNSS: total nasal symptom score; TOSS: total ocular symptom score; NTSS: night-time symptom score; SD: standard deviation.

Parameter	Visit 1 (Mean ± SD)	Visit 5 (Mean ± SD)	p-value
TNSS	6.73 ± 2.28	0.86 ± 1.11	0.005
TOSS	5.58 ± 2.44	0.61 ± 0.87	0.005
NTSS	4.99 ± 2.29	0.37 ± 0.60	0.005

The study did not include objective biomarkers or imaging assessments to evaluate disease regression. Outcomes were limited to validated clinical symptom scores and safety assessments.

Global assessment of efficacy and tolerability

At week 13, global assessment of efficacy showed that the majority of patients rated overall symptom improvement as good or very good on the 4-point scale. Investigator assessments were consistent with patient-reported outcomes. Tolerability was rated as good or excellent in most patients on the 3-point scale, with no reports of poor tolerability

Safety analysis

Safety was assessed in all 200 patients who received at least one dose of the fixed-dose combination of levocetirizine hydrochloride 5 mg and montelukast 10 mg. The overall incidence of adverse events (AEs) was low, with the majority of patients (n=172; 86.0%) reporting no AEs throughout the study duration. Among the 28 patients (14.0%) who experienced at least one adverse event, all events were mild in nature and resolved without the need for discontinuation of treatment. The AEs include fever, observed in 8 patients (4.0%), followed by headache in 6 patients (3.0%) and gastric problems in 5 patients (2.5%). Other reported events included cold (2.0%), anxiety and chest pain (0.5%), cough (0.5%), discomfort (0.5%), increased sleep (0.5%), and loss of appetite (0.5%). No serious adverse events or deaths occurred during the study, and no new safety concerns were identified. These findings support the favorable safety and tolerability profile of the fixed-dose combination treatment in patients with seasonal allergic rhinitis. The frequency of adverse events is presented in Table 4.

**Table 4:** Frequency of Adverse Events. Adverse events (AEs) were recorded for all patients who received at least one dose of the fixed-dose combination of levocetirizine 5 mg and montelukast 10 mg. All reported AEs were mild in nature and resolved without intervention. No serious adverse events or treatment discontinuations occurred during the study period. The majority of patients (86%) reported no adverse events, indicating a favorable safety and tolerability profile.

Adverse events	Frequency	Percentage (%)
Anxiety and chest pain	1	0.5
Cold	4	2.0
Cough	1	0.5
Discomfort	1	0.5
Fever	8	4.0
Gastric problems	5	2.5
Headache	6	3.0
Increased sleep	1	0.5
Loss of appetite	1	0.5
No adverse events	172	86.0
Total	200	100.0

## Discussion

This study demonstrates that the fixed dose combination (FDC) of levocetirizine hydrochloride 5 mg and montelukast 10 mg is effective and well-tolerated in the treatment of seasonal allergic rhinitis (SAR) in adult Asian patients. At baseline, the high prevalence of sneezing (100%), rhinorrhea (97%), nasal congestion (95.5%), and nasal itching (88%) observed in this study reflects the classical symptom burden reported in SAR cohorts worldwide. Similar symptom distributions have been reported by Bousquet et al., who identified sneezing and rhinorrhea as nearly universal symptoms in untreated allergic rhinitis, with nasal congestion being the most persistent and treatment-resistant manifestation [27].

The pathophysiology response of allergic rhinitis begins when allergen-specific immunoglobulin E (IgE) attaches to mast cells, which subsequently release histamine, resulting in sneezing and rhinorrhea. This is followed by a cascade of inflammatory mediators such as prostaglandins, cytokines, and leukotrienes that cause persistent nasal congestion and delay the symptoms [14]. The fixed-dose combination (FDC) was effective in controlling both early-phase symptoms (e.g., sneezing, rhinorrhea) and late-phase symptoms (e.g., nasal congestion and itching).

The significant reduction in TNSS observed in our study, from a mean of 6.73 at baseline to 0.86 at week 13, indicates a marked reduction in nasal symptoms in the majority of patients. These findings are consistent with a systematic review and meta-analysis demonstrating superior nasal symptom control with combined antihistamine and leukotriene receptor antagonist therapy compared with antihistamine monotherapy [28]. The statistically significant clinical improvement observed is likely due to the dual-action profile: histamine-mediated pathways [15]. Similarly combination of montelukast and levocetirizine emerges as a synergistic combination that targets two inflammatory pathways and enhanced efficacy, as stated by Bruce Chandler May et al [16], supports our findings.

Ocular symptoms were highly prevalent at baseline in our study, with tearing (90%) and eye redness (87%) commonly reported, consistent with previous clinical descriptions of allergic rhinitis, where ocular involvement frequently accompanies nasal symptoms [29]. The marked reduction in TOSS over the treatment period aligns with evidence that histamine plays a central role in allergic conjunctival inflammation and that second-generation antihistamines such as levocetirizine provide rapid and sustained ocular symptom relief [30]. A study by Yogesh D et al. stated that levocetirizine provides effective symptom management for the entire day and has minimal sedative effects, leading to better sleep and daily functioning without significant daytime drowsiness [17].

Night-time symptoms showed a clinically meaningful improvement in our study, with NTSS decreasing from 4.99 ± 2.29 at baseline to 0.37 ± 0.60 at week 13, reflecting improved sleep quality and reduced morning nasal congestion This is in line with pivotal study by Meltzer et al. which supports our findings that montelukast significantly improved the NSSS, a clinically important outcome in SAR studies [18]. On the other hand, Montelukast’s ability to decrease airway resistance and nasal congestion could further explain the nocturnal alleviation that our cohort experienced [19].

The ocular symptom burden, although often overlooked, improved significantly during the study period. Levocetirizine is likely responsible for the rapid resolution of eye redness and tearing through its histamine-blocking effects. Our findings are further supported by a recent meta-analysis from Krishnamoorthy et al., which stated that montelukast and an oral antihistamine taken together are more effective at managing SAR symptoms than either one alone [20]. The safety profile of the FDC was positive, with 14% of patients experiencing mild, self-limiting adverse effects. There were no discontinuations or serious adverse outcomes. These findings align with the long-established safety data of both agents, individually and in combination, as noted in references [21-23].

Limitations

This study was strengthened by using an adequately powered sample size along with patient-centric symptom assessment tools and adherence verification with rigorous compliance monitoring. However, the open-label, single-arm design and absence of a control group limit causal inference, and the ethnically homogeneous study population may restrict the generalizability of the findings.

The single-arm, open-label design without a comparator group limits causal inference. The absence of objective inflammatory markers restricts the evaluation of disease modification. Additionally, the relatively homogeneous study population may limit generalizability. This study did not assess objective markers of allergic inflammation or structural disease progression. Therefore, conclusions are limited to symptomatic improvement rather than disease modification. Future studies incorporating objective inflammatory biomarkers or nasal airflow measurements would provide a more comprehensive evaluation of therapeutic impact.

## Conclusions

The combination of levocetirizine hydrochloride 5 mg and montelukast 10 mg (L-Montus) showed statistically significant changes in efficacy and was well-tolerated for controlling seasonal allergic rhinitis (SAR) in adult Asian patients. Nasal, ocular, and nocturnal symptoms were significantly reduced in patients with the use of the validated TNSS, TOSS, and NTSS. The regimen provided steady symptom control during the day and caused minimal sedative effects, thus improving the quality of sleep and daily functioning without any clinically significant daytime drowsiness. The therapeutic benefit is probably because of the dual mechanism of action, which is inhibition of the histamine-mediated early-phase reactions and leukotrienes-mediated late-phase inflammation. The tolerability profile was acceptable as evidenced by safety data. In this regard, the levocetirizine-montelukast fixed-dose combination can be regarded as a potential alternative to the treatment of seasonal and perennial allergic rhinitis.
